# Precision Livestock Farming in Swine Welfare: A Review for Swine Practitioners

**DOI:** 10.3390/ani9040133

**Published:** 2019-03-31

**Authors:** Madonna Benjamin, Steven Yik

**Affiliations:** 1 Department of Large Animal Clinical Sciences, College of Veterinary Medicine, Michigan State University, 736 Wilson Rd, East Lansing, MI 48824, USA; 2 Department of Electrical and Computer Engineering, College of Engineering, Michigan State University, 428 S Shaw Ln, East Lansing, MI 48824, USA; yiksteve@egr.msu.edu

**Keywords:** swine, welfare, critical criteria, precision livestock farming, practitioner, remote monitoring, CSIA

## Abstract

**Simple Summary:**

The increasing implementation of technological advances originally developed for video gaming (PlayStation, Xbox) is helping to progress livestock production so that it is both more efficient and more focused on the welfare of the animals. Such advances are necessary to ensure that innovations can emerge from applications using cameras, microphones and sensors to enhance the farmers’ eyes, ears and nose in everyday farming. This technology for remote monitoring of livestock, termed precision livestock farming, is the ability to automatically track individual livestock in real time. The goal of this review is to apprise swine veterinarians and their clientele on precision livestock farming with a general introduction to the technology available, a review of research and commercially available technology and the implications and opportunities for swine practitioners and farmers. Drawing from pig welfare criteria in the Common Swine Industry Audit, this review explains how these applications can be used to improve swine welfare within current pork production stakeholder expectations. Swine veterinarians and specialists, by virtue of their animal advocacy role, interpretation of benchmarking data, and stewardship in regulatory and commodity programs, can play a broader role in facilitating the transfer of precision livestock farming and technology to their clients.

**Abstract:**

The burgeoning research and applications of technological advances are launching the development of precision livestock farming. Through sensors (cameras, microphones and accelerometers), images, sounds and movements are combined with algorithms to non-invasively monitor animals to detect their welfare and predict productivity. In turn, this remote monitoring of livestock can provide quantitative and early alerts to situations of poor welfare requiring the stockperson’s attention. While swine practitioners’ skills include translation of pig data entry into pig health and well-being indices, many do not yet have enough familiarity to advise their clients on the adoption of precision livestock farming practices. This review, intended for swine veterinarians and specialists, (1) includes an introduction to algorithms and machine learning, (2) summarizes current literature on relevant sensors and sensor network systems, and drawing from industry pig welfare audit criteria, (3) explains how these applications can be used to improve swine welfare and meet current pork production stakeholder expectations. Swine practitioners, by virtue of their animal and client advocacy roles, interpretation of benchmarking data, and stewardship in regulatory and traceability programs, can play a broader role as advisors in the transfer of precision livestock farming technology, and its implications to their clients.

## 1. Introduction

By 2050, the global human population is projected to be over 9 billion, consuming 50–60% more food [1] compared to the present consumption patterns. Notwithstanding inequalities, globally, the population is becoming richer and will tend to choose livestock products, preferring meat, milk and eggs over staple tubers and grains [2]. Without modifying our current consumption practices, there will be significant increase in food insecurity globally. Godfray and Garnett write that one solution to this security issue is sustainable intensification with the following framework: (1) a need for increased production of food, (2) that food must come from existing agricultural land, and (3) a broad range of tools and production methods to be considered [2]. Within this framework, livestock intensification will increase animal density and lower the stockperson per animal ratio [3]. As such, animal welfare may be one potential trade-off in favor of sustainable intensification. Broom [4] wrote that people consider that they have obligations to animals they use, whether for companionship, transportation or food production, and expect care of individual animals. While economists infer meat production will increase, there are societal expectations that animals used for meat will be treated humanely and individually.

To address consumer and public concerns of how pigs are currently raised in commercial production, Pork Quality Assurance^®^ Plus (PQA Plus^®^) introduced the Common Swine Industry Audit—Pork Checkoff, a third party on-farm auditing program to provide pork chain stakeholders with a consistent, reliable, and verifiable system of on-farm swine welfare and food safety [5]. Under Common Swine Industry Audit (CSIA) welfare assessment, twenty-seven audit criteria are divided into critical failure criteria, animal and resource-based measures, and pre-harvest food safety. Critical failure criteria include observation of animal abuse or compromised animals that are not euthanized in a timely manner [5]. If these criteria are found to be unacceptable by the auditor, the site will automatically fail the audit (Common Swine Industry Audit—Update 2019) with potential loss of market for the farmer. Animal measures of well-being include recorded assurances that pigs are assessed daily to identify and treat compromised pigs such as low body condition, severe lameness, tail-biting lesions, rectal and uterine prolapses, umbilical hernias, open wounds and scratches. Current training for farmers, assessors, and CSIA auditors includes visual subjective scales for scoring body condition and lameness. PQA Plus^®^ and the CSIA use a simple diagram on a scale of 1 to 5, with 1 = emaciated, and 5 = very fat, to subjectively identify body condition. While most evaluators can consistently discern a 1 and 5 score, scores 2, 3 and 4 are less consistent [6]. Pigs demonstrating the most severe lameness (i.e., score 4 = not weight bearing on the affected limb(s) when either standing or walking) are identified and scored during the audit. In general, on-farm lameness evaluation is inconsistent among assessors [7]. Compounding this complexity, the current stockperson to sow ratio in commercial farrowing units is approximately 1:300 [8], requiring the stockperson to make subjective health assessments in large groups of animals in a short period of time. With increasing herd size and decreasing workforce availability, precision livestock farming can combine sensors and complex data to provide a simple score that is meaningful and understood by all farm workers, ensuring optimal health, productivity, and pig welfare [3].

While there are publications which address precision livestock and welfare [9,10,11,12,13,14,15], the aim of this review is to provide pig practitioners a general description of the tools and applications of precision livestock farming for pigs, so they can apply this basic knowledge in collaboration with engineers and technology developers in consultation with their clientele. For this review, the term swine “practitioner” encompasses professionals, such as veterinarians, animal scientists and geneticists, who have expert knowledge of both pigs and their producer clientele. The review covers terminology and an introduction of algorithms and machine learning, and describes research and examples of commercial applications in remote animal recognition and monitoring. Using Common Swine Industry Audit criteria, it discusses welfare challenges faced by the swine sector and describes how precision livestock farming can improve pig welfare and productivity. Finally, discussed are the opportunities for swine practitioners as welfare experts and consultants to apply and integrate precision livestock farming.

## 2. Precision Livestock Farming: An Overview

“Monitoring animals within groups is challenging, even for the best herdsmen” NPB—Animal Science Committee” [16]. Precision livestock farming implies automated remote detection and monitoring of identified individuals for animal health and welfare using real-time analysis of images, sounds, tracking data, weight and body condition, and biological metrics in livestock [15,17,18]. With that, there is the capacity for early detection of illness or physiological status at the farm level. While not a new science [19], the information, applications, and availability of precision livestock farming has greatly increased due to computer science expertise and inexpensive sensors off-labelled from the video gaming industry (Xbox, PlayStation^®^, etc.) combined with the growing computer capacity for capturing and processing data [13,15,20].

### 2.1. Sensors and Animal Recognition

In order to better understand how current technology may impact the world of precision livestock farming, a basic understanding of remote monitoring sensors, the process of algorithm development and machine learning is required.

Remote sensors such as cameras, microphones, thermometers and accelerometers monitor or capture information such as images, sound, heat or motion from groups or individual animals. The data from the sensors, stored in external drives or sent directly to a processing node (analogous to transferring photos from a digital camera to a computer) are then processed by algorithms. An algorithm is a formula, or step-by-step set of operations, used to solve a specific problem or class of problems. A programming algorithm is a computer procedure that tells a computer precisely what steps to take to solve a problem utilizing inputs to determine the outputs. Programmers initiate the process by writing the algorithm that instructs the computer how to perform the specific operations necessary to solve a problem. An algorithm’s value to farmers is dependent on its ability to transform the sensor data or “feature variable” to a biological outcome. Examples of feature variables include percentage of time pigs are lying down to determine the biological outcome “lameness” or the number of coughs to detect the biological outcome “respiratory disease”. Machine learning is a family of computational methods that allows an algorithm to program itself using large sets of examples. Because the computer “learns” from these example sets of existing data, a system can become highly adept at processing and analyzing large data sets to track variables and produce estimates at a rate that would not be possible for humans or traditional statistical methods [21]. Taken together, data from remote monitoring sensors is combined with individual animal identification, referenced observations and production data, and then integrated in algorithms to provide credible information and alerts regarding pig welfare, health and productivity [22,23,24,25].

#### 2.1.1. Cameras (2D and 3D)—Behavior and Physiology

**Figure 1:**
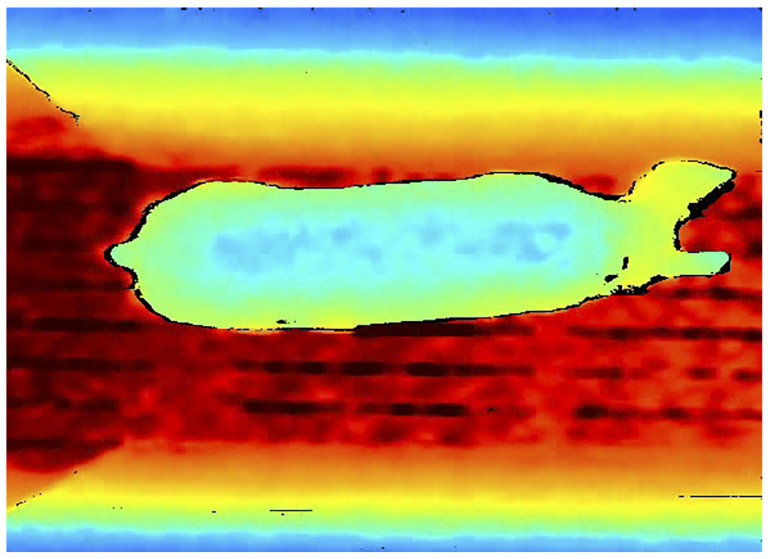
Depth camera sample image containing a sow.

Image analysis translates the acquired images into indices of distribution (animal location and proximity) and activity (animal position and movement) [26]. Imaging in pigs has been used to estimate pig weight [27,28,29], aggressive behavior [30], walking patterns [31], sow posture and behavior during lactation [32]. Image analysis research using two-dimensional (2D) cameras, provided digital information such that researchers were able to monitor and estimate pig growth rates within 1 kg [33,34]. However, 2D camera sensors require adequate ambient lighting and contrasting background—such as a white pig on dark cement. Three-dimensional depth-based sensors (3D camera) such as Microsoft Kinect (Microsoft, Redmond, Washington) and Intel^®^ RealSense™ (Intel, Portland, Oregon) cameras are equipped with a high-definition camera, an infrared illuminator and time-of flight (ToF) depth sensor that produces color. Infrared is of particular importance during low lighting or observing nocturnal behavior [27,35] and depth sensors are important to determine the proximity of the animal to the camera [36]. ToF depth technology sends a pulse of infrared light from an LED multiple times a second, and then records the delay between the pulse and its return to each pixel [13]. Figure 1 shows an example of a depth image with red being furthest and blue being closest within the image. This is useful when capturing the variations of depth to construct a “topological map” of a pig for 3D geometry calculation. Depth-based cameras are advantageous due to their low cost (less than $180 USD), ability to handle large data sets [37], low power requirements and ability to adapt to variable light and background conditions [38]. Current 3D cameras require a retrofitted cover to protect the sensors from environmental assaults such as ammonia, moisture, dust and insects.

#### 2.1.2. Microphones—Sound

Relatively simple microphones convert noises into electrical signals that may be processed in computers with the intent of detecting, classifying, and localizing specific acoustic events such as indications of stress or illness [39]. For example, high frequency calls of pigs have been linked to stressful situations [40] and “coughs” could be linked to respiratory diseases and thus to their welfare [41]. Therefore, with the implementation of sensor technology, microphones and vocalizations could become an automatic daily measure.

#### 2.1.3. Thermistors and Infrared Imaging—Temperature

Temperature monitors using a contact measuring media typically utilize thermistors embedded in a data logger or ear tag sensor. The sensor has direct contact with the tissue to take temperature measurements and provide temperature accuracies to 0.1 C [42]. Infrared technology does not require any contact with the animal, allowing remote measuring. The physical basis of infrared technology is that any object that has a temperature above absolute zero (0 K) emits infrared radiation and the temperature of the object determines the wavelength of radiation emitted. The conversion of radiant heat into a computer-generated color image is done with a process called thermal imaging. Infrared cameras measure physiological and pathological processes related to changes in body temperature [43] as a non-invasive, instantaneous method [42,44]. Peripheral temperature readings depend on core temperature, environmental conditions and the peripheral blood system regulation. In higher ambient temperatures, thermoregulation directs increased blood flow to the skin tissue resulting in higher surface temperatures [42]. In older pigs, fat insulates the body core—at low ambient temperature, skin temperatures are lower. Surface areas such as the tissue behind the ear, or mammary tissue are devoid of hair or insulation, and reflect better an adult pigs’ core body temperature [42]. Whereas, due to their lack of body fat and insulation, the skin temperature of newborn piglets is a “thermal window” to their core temperature [45].

#### 2.1.4. Accelerometers—Motion Tracking

Among the most promising technologies for monitoring livestock behavior are wearable sensors containing accelerometers. An accelerometer is an electromechanical device used to measure accelerating forces. Forces can be static (e.g., pig is lying down) like the continual force of gravity or acceleration due to movement (e.g., pig is walking). Movement generates a stress on microscopic crystals housed within the accelerometer and creates voltage. The sensors interpret the amount of voltage to determine velocity of the movement and orientation. A tri-axial accelerometer accumulates three-dimensional information (*x*, *y* and *z* axis) and measures the earth’s gravitational pull by determining the angle at which the device (e.g., ear tag, neck collar) is tilted in addition to measuring acceleration forces.

### 2.2. Livestock Identification

As large-scale pig production continues to grow, a prerequisite for the linkage of animal data to precision livestock farming systems is through animal identification systems that are automated and affordable for the farmer [20]. Individual identification methods, either currently used in the swine industry or research, include radio frequency identification, optical character recognition, and facial recognition.

#### 2.2.1. Radio Frequency Identification (RFID)

A well-established technology for swine identification, health purposes and management on pig farms is the RFID chip [46,47,48,49]. The device is primarily implanted in ear tags; it stores information such as animal and farm records. The radio wave (low, high or ultra-high frequency) is the medium of communication between the transponder circuit within the tag and an RFID reader to wirelessly read and write data. The working principle of such a device is that when an RFID tag comes within range of an RFID reader, it receives a signal. Furthermore, a second radio frequency signal is induced, carrying data that travels to the reader [48]. These data can be stored and analyzed later, or the RFID chips can be used immediately to identify individual animals.

Low frequency (LF)—RFID is a valuable component of groups housing electronic sow feeders; these small feeder enclosures fit one sow at a time and dispense a specific limited diet to the sow while also collecting identification data and feeding frequency via RFID [50]. Infrequent visits of certain sows could raise an alert to producers so that health issues or undesirable social behaviors may be addressed early in their progression [51]. Nevertheless, LF-RFID has two major disadvantages: a low read range (<1 m), and the inability to identify more than one animal [52] within range. In order to track multiple animals at a greater range (3 to 10 m), researchers have investigated the commercial feasibility of Ultra-High Frequency (UHF) readers [49,53]. While promising, the UHF-RFID transponder ear tags are sensitive to interference from ear tissue, leading to false registrations [49,54], thus requiring further research to adapt the technology for consistent use in pig identification. Other drawbacks to RFID include loss or attrition of tags, pain and stress of the animal during tagging and required removal from the animal prior to slaughter processing.

#### 2.2.2. Optical Character Recognition

**Figure 2:**
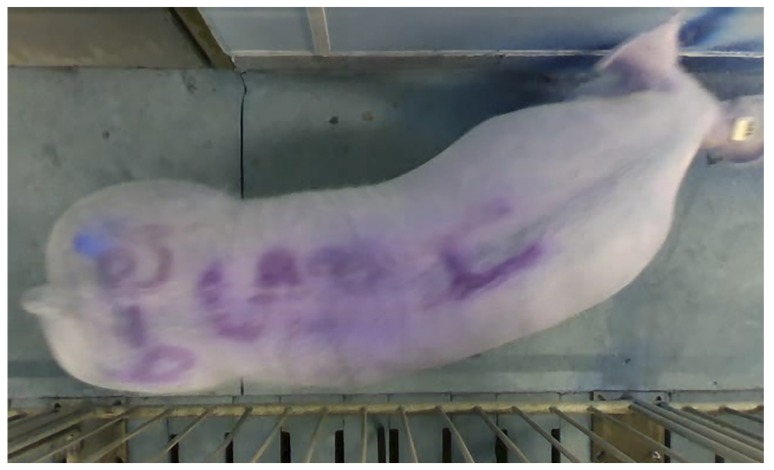
Painted numbers and ear tag for optical character recognition on a sow.

A low-cost identification system, optical character recognition, is the recognition of printed, stamped, or written text characters (e.g., license plates, barcodes, QR codes) by a computer. In pig production, optical recognition includes characters on ear tags or painted symbols and numbers (Figure 2). Optical character recognition is performed with a digital camera and data is developed with machine learning to provide remote identification [36]. Depending on the clarity and color of the markings, there is the capacity to identify large permutations of animals and, with the exception of optical character recognition on tags, the identification may not need to be removed from pigs prior to slaughter [55]. When the characters are painted, visual identification patterns can fade within a day and when pigs lie close to or on one another, pattern recognition is occluded [56].

#### 2.2.3. Facial Recognition

**Figure 3:**
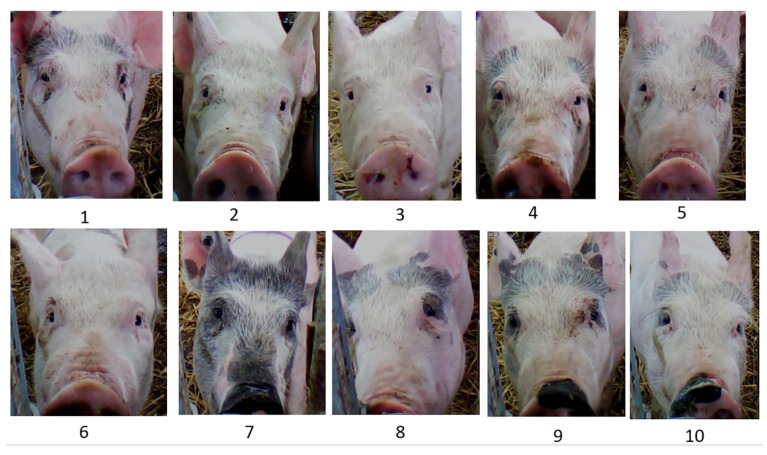
Set of images used for facial recognition training.

An example of marker-less individual pig identification is facial recognition, initially developed for human identification, monitoring, and surveillance purposes [57]. Using methods known to efficiently recognize human faces, Wada [58] examined frontal photos from 10 pigs and achieved 77.0% recognition from the full face and 97.9% when the reading was focused on the eye region. Hansen [47] used digital photos taken from a camera mounted on a water drinker and developed a program that differentiated 10 pigs (Figure 3) [47], with 96.7% accuracy. Hansen’s algorithm recognized pigs from three regions: the snout and wrinkles above the snout, prevalent marking at the top of the head, and the eye regions. This technology is promising due to the speed of recognition (620 images/sec) and the application of human recognition algorithms for pig faces.

### 2.3. Hardware and Software Comparison Summary

As a holistic summary, Table 1 illustrates possible features and fallbacks of sensor devices and specific examples of applied applications.

### 2.4. Mobile Applications, WiFi and Bluetooth

As today’s age of technology keeps expanding, the current networking infrastructure limitations for the farm industry poses challenges. As most farms are in rural regions, WiFi and mobile services are not within, or do not supply, sufficient networking abilities for newly connected technologies [59].

Note that not all precision livestock farming devices will require internet connectivity. Sensors can be used in isolation where encrypted data can be collected from various sites within the farm system, compiled, and sent to a local computational system for processing to filter unnecessary data. Then, the utilization of mobile applications such as smartphones and tablets can be used for convenient display of results or alerts via WiFi or Bluetooth.

### 2.5. Precision Livestock Farming Technology Design and Implementation

The development of a new technology for precision livestock farming requires a deep understanding of the specific task to identify what type of sensor best suits those needs. There are various ways in which to design and implement such new devices. Along with the supporting infrastructure that is required, several example implementations can be found in Table 1. Single sensor designs are easier to integrate and develop, but are restricted within sensor capabilities. Implementations which include multiple sensor types come with an added benefit of higher accuracy and robustness, but at the cost of the design complexity of sensor fusion to create a joint signal for processing.

Validation of data and how to identify the accuracy or precision of such measurement or prediction requires background knowledge in statistics and probability [60]. In a practical setting, human observations can be taken from well-trained (experts) individuals and can be compared to device results to quickly assess performance. For fine accuracy, statistical methodologies such as least squares and maximum likelihood estimation can be performed on the data to evaluate accuracy. Standard engineering design processes such as preliminary hazard analysis, design failure mode and affect analysis, and design reviews should be utilized [61] especially within farm settings where possible hazards are not accounted for, resulting in performance loss. Having close communication with farms and being able to test and prototype designs will help identify what design constraints are necessary for a successful device.

## 3. Welfare Challenges in the Swine Industry

Consumers expect their animal-derived food to be produced with respect for the welfare of the animals. Subsequently, a number of standards and measures have been developed to assure the public that livestock have received proper treatment [62]. Under the Common Swine Industry Audit, all stages of pig production are assessed using animal-based measures [5]. In the next section, this paper will focus on a general description of individual and group pig welfare challenges such as lameness, body condition, prolapse, pig comfort, antagonistic behavior and recognition of illness.

### 3.1. Lameness

Lameness, a condition that inhibits or modifies the gait of an animal, is a clinical sign associated with a range of conditions such as claw lesions, trauma, osteochondrosis, fractures, skin lesions and arthritis [63,64]. Within-herd prevalence of lameness is high (ranging between 8–16%) [64] and results in economic losses from unplanned culling of sows [65]. Sows removed for lameness had 1.4 fewer litters than the average sow [66]. Moreover, the association of lameness with pain [67,68] diminishes optimal welfare in pigs.

The CSIA identified lameness as a significant welfare issue in commercial production and severely lame pigs are a parameter of critical failure criteria [5]. Pairis-Garcia and Moeller wrote “…application of objective methodologies to assess lameness consistently on farm and identify mildly and moderately lame populations of pigs are also needed to improve pig welfare prior to the condition ending in severe lameness”.

### 3.2. Body Condition

Body condition scoring (BCS) is defined as the physical assessment of body composition to evaluate quality of diet [69]. There is particular value in retrieving predictive indicators of body condition due to the correlations with lameness and shoulder sores [70,71]. Studies in dairy cattle proposed temporal relationships between low body condition and lameness where cattle with BCS <2 were at greatest risk of lameness, and cows that suffer a greater decrease in BCS, had a higher probability of becoming lame and a lower probability of recovering in the next 15 days [72]. Survey data showed that sows culled from the breeding herd for BCS and lameness had less (*p* < 0.01) back fat. Furthermore, thin sows, as measured by body weight and low back-fat depth, are more likely to be culled from the herd [73]. A quantitative caliper to measure the angularity of sows was developed on the premise that as a sow loses muscle and fat, her back becomes more angular [6]. Effective reading of the caliper requires sows to have limited movement and that human placement of the caliper, centered over the spine and at the last rib, is achieved.

### 3.3. Prolapse Syndrome

The incidence rate in sow uterine, vaginal and rectal prolapses has been increasing across the industry from an average of 1.0% in 2013 to 3.0% in 2016 [8]. Welfare requirements include immediate euthanasia of pigs; a uterine or vaginal prolapse results in both sow mortality and loss of unborn piglets. As prolapses, based on organ protrusion, are easily identifiable by the stockperson, the score is quantitative and therefore there is a reasonable speculation that, with enough imaging, machine learning may detect causal variables such as sow phenotype or posture.

### 3.4. Welfare at the Group Level

At the group level, ill and injured individuals represent a vulnerable population with unique needs and preferences [74]. During CSIA audits, animal-based measures include daily observations to monitor pig comfort and detect abnormal behavior or clinical indications of disease, injury or pain [5]. Millman notes that sickness behavior includes altered behavioral responses such as shivering, huddling and resting, changes in social interaction, reduced feeding and drinking [74]. Cook measured spatial distribution of piglets post-vaccination, noting more periods of huddling associated with a febrile response [75]. Assessment of pigs unaware of human presence, assists in observing their current behavior patterns. Pig comfort, is indicated by thermal comfort behavior of pigs as too cold, comfortable, or too warm based on lying position and posture within the pen [5].

Negative pig-directed behaviors include oral events such as ear, flank and tail biting. Tail biting causes wounding as well as partial or full amputation of the tail [76]. Tail biting has many contributing factors including a stressful environment and excessively aggressive social feeding behavior [76,77]. Slaughter surveys of pigs with bitten tails showed an increased risk of lower weights, respiratory lesions, locomotory problems, abscesses and arthritis [78]. Further consideration of tail biting includes timely euthanasia requirements of pigs with open sores (e.g., tail and flank biting) that are unlikely to recover from treatment after two days.

Monitoring and controlling some behavioral responses to population indicators of illness could improve individual pig welfare and animal-based measure outcomes. While sound detection is not an animal-based measure in the CSIA, coughing, a frequent symptom of respiratory illness within a population, could be used to substantiate treatment regimens and reduce individual pig morbidity.

## 4. Summary of Remote Monitoring Technology Applications for Swine

In the next section, this paper will focus on a general description of individual and group pig welfare challenges such as lameness, body condition, prolapse, pig comfort, antagonistic behavior and recognition of illness and corresponding research and remote monitoring applications within precision livestock farming.

### 4.1. Lameness and Mobility

Lame sows can be expected to behave differently due to physically reduced locomotion, pain or general discomfort and sickness behavior [64]. In groups, sows with non-resolved lameness were observed to move and stand less, lie down more, and were in contact with the wall more than healthy control sows [79]. These differences in behavior could be interpreted as signs of pain or as a way of seeking shelter and isolation from the group. Unfortunately, with less employees overseeing more animals, lameness is often undetected until it is moderate to severe and, if in late gestation, economics encourages lame sow retention through lactation.

Techniques to automatically score lameness include pressure mat or force plate systems, imaging and accelerometers. Force plates [80] (Matscan^®^ and SowSIS (Tekscan, South Boston, MA; Institute for Agricultural and Fisheries Research, Melle, Belgium)) and pressure-sensing mats (GAITFour^®^ (CIR Systems, Inc., Havertown, PA, USA)) are reliable technologies that can identify abnormal or asymmetric gates in lame pigs [81]. Force plates have been used to measure pressure distribution of claws [82], weight distribution on all four legs of sows [83], and leg loading and weight shifting [84]. The GaitFour^®^, an electronic pressure mat and software assesses lameness through measures including maximum pressure, stride length, stance time, stride time, and activated sensor count per foot in both sows [85] and weaned pigs [81]. Pressure mat or force plate measures provide referenced standards for lameness [85]; they have been incorporated into electronic sow feeders and breeding or gestation crates, but they require specific and complex installations in a swine barn [86].

Lameness has been classified through other variables such as reduced walking speed, shorter or uneven strides, and swaying from side to side [87]. These visual features can be detected through motion tracking and topological analysis of these animals. Based on the success of image analysis in dairy cattle to predict lameness [88,89], it is likely that an objective lameness detection system using imaging will be developed for sows. In our research, using motion tracking between frames from consecutive images of a video, a lateral motion path is calculated and compared to the actual forward movement of each sow (unpublished data).

Research has demonstrated efficacy of accelerometers attached to the leg of sows to detect posture and stepping behavior, standing duration, latency to lie down after feeding, and step frequency when feeding [84,87,90] and from pre-parturient nesting activity, detected the onset of farrowing [91,92]). As sows would chew on devices fitted elsewhere, data sampled from ear tags seems to be the most sustainable and commercially suitable method [91]. Applying ear tag accelerometers to sows, researchers determined that while the ear is the most decoupled body part of the locomotory system, a prediction of lameness was demonstrated when comparing variables’ high activity (distance walked) and rest phases (lying down). High activity (distance) was lower in lame sows and detected 14 days prior to signs of moderate lameness [86,92]. Another study [93] supported the sensitivity of accelerometers to detect static behavior such as time that an animal spends lying down (94.3%), but found other behavior variables such as time spent standing (66.9%) and time walking (68.4%) were lower in accuracy.

### 4.2. Pen Level Activity Monitoring—Nursery and Grower Pigs

Applications to improve welfare and automate pig monitoring include video images to measure pen level activity [94,95] such as antagonistic behaviors, chasing, tail and flank biting, fighting, head-to-head knocks between pigs [30,96,97,98,99]. Sensor data differentiates lying patterns of pigs (thermal comfort behavior) [100,101], standing pigs from moving pigs [38], and lateral from sternal recumbency [102]. Depth imaging tracking has shown promise to monitor pig location, eating, drinking and aggression interactions between pigs [55,99]. Currently, it is difficult to use sensor tracking for individual pig movement in a large group, due to the nature of pigs to pile and lie close.

One approach to pen monitoring was the use of 3D cameras and machine learning to detect pig activity and provide an automatic warning of tail biting “outbreaks”. As tail docking is banned in the EU, researchers monitored the non-docked tail posture of pigs [103]. This study noted that the proportion of pigs with low tail posture was highest one week prior to outbreak and a greater proportion of injured pigs were associated with low tail posture.

#### 4.2.1. Infrared Thermography

Various studies have shown that infrared thermography is a means to non-invasively detect dissipation of heat in individual animals or specific regions of the body for the purpose of rapidly detecting diseases such as mastitis [44,104], locomotion disorders [105,106,107], and respiratory disease [108] in bovine. Skin measurement sites for pigs using infrared thermography, with the highest correlation to body temperature, are the ear base, eyes and udder [42]. Infrared thermography has been used to determine individual illness in groups of piglets. Vaccination is known to initiate high skin temperatures and huddling responses were observed up to 20 hours post vaccination in a group of piglets [109].

#### 4.2.2. Sound Detection

Sound recordings can be used with an algorithm for vocal analysis to detect heat stress [110] and high frequency “screams” of pain [25] as a consequence of tail biting or fighting. Algorithms also distinguished between infectious productive coughs and non-infectious, non-productive coughs (ammonia or dust) from differences in the acoustic variables [111,112]. A commercially available sound detection package, Soundtalks^®^ (Leuven, Belgium) recognizes sounds in a localized area, enabling treatment for respiratory disease and ventilation changes at a pen level, rather than the entire barn [41]. Sound detection can be inhibited if the barn is noisy or there are insufficient microphones. The ability to consistently distinguish between stress-related and normal vocalizations would be beneficial as pigs, in a way, would be able to “speak for themselves” about their welfare.

#### 4.2.3. Live Weight, Body Condition and Physiology

Live weight, shape, growth and body composition are crucial factors in the management of swine production because individual pig weight and growth affects the herd in factors such as barn flow and space allowance, and audit parameters [27,113]. An animal-based measure of CSIA includes a spatial (mass) allowance such that 90% of pigs can lie down at once in group pens and that sows can lie down fully in stalls. Studies have shown promise and commercialization in the area of extracting the 3D shape of pigs for automatic mass and weight estimations [35,114,115,116].

#### 4.2.4. Wireless Sensor Networks

**Figure 4:**
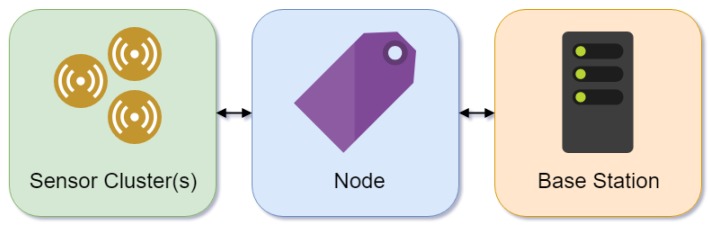
Wireless sensor network structure.

Wireless sensor networks (WSNs) often consist of a sensor(s), node and base station (Figure 4). Generally, low-cost and low-power, multifunctional sensors such as a thermistor, accelerometer and battery are clustered in one node. For pig production, the sensors, such as accelerometers, are encapsulated within an ear tag (node) which holds the sensor package for communication. The node sends information to the base station which provides connectivity to the server or might perform computational tasks [117].

An example of a commercial WSN is “Remote Insights—Wireless Asset Management System” (Remote Insights, Minneapolis, MN). According to the patent, accelerometer and temperature gauge sensors housed in the node are sealed to prevent entry of moisture or dust. The ear tag node (beacon), communicates its unique identification to a gateway or base station via cellular, satellite, WiFi or Ethernet [118] and includes immediate visual communication with the farmer using diodes that emit alerts or behavior patterns such as locomotion disorders [119].

## 5. Precision Livestock Farming—Practitioner and Consultant

Paraphrasing Ramirez and Karriker [120], a successful swine practitioner is one who solves problems, creates opportunities and promotes the financial success of their clients. Swine veterinarians have worked closely with farmers to shift disease management practices and have long promoted the value of swine management software and analytics programs (e.g., PigCHAMP, PIGKnows, MetaFarms) to provide key production indices (e.g., pig mortality, pigs produced, market weights and feed conversion) in herd or batch reports [121,122]. Data entries provided by the farms include descriptive information (e.g., farrowing dates, number of piglets born) and diagnostic information (e.g., reason for death, response to treatment) [123].

Alternatively, using sensors to digitize physiological variables, animals are monitored constantly and make generated data predictive and prescriptive [124]. This data can be utilized to predict trends and behavior patterns and support decision-making [125] without information bias [59]. One consideration is a method for detection of lameness (yes/no) post analgesic treatment [126]. Consequently, pig welfare can be improved if farmers can specifically evaluate the economic outcomes of mitigation practices.

### 5.1. A Technical Role of Swine Practitioners

Animal welfare is both an ethical driver with economic consequences and an economic driver that carries moral weight [9]. The most obvious way in which welfare and economic efficiency go hand in hand is through reduction in mortality and morbidity. The most humane act to welfare problems such as perforated hernias, uterine prolapse, pigs that are severely injured or unable to stand or walk, and without likelihood of response to treatment, is timely euthanasia. Attributing variables to these problems could realistically be associated with animal phenotype, pen activity or posture and early detection could improve both profits and welfare.

Practitioners not only determine disease occurrence and risk factors but also the prevalence and the value of diagnostic results within complex farms and systems [74]. Moreover, while not specifically trained in applied ethology, swine practitioners, through their training and multiple on-farm visits, recognize normal from abnormal physiology and risk factors of poor welfare. The goal of precision livestock farming technology is to provide detection and subsequently early indicators of problems that identify individual animals or specific groups, that need attention. Swine practitioners and their clientele must be confident that the alerts are both highly sensitive and specific to assure detection and avoid unnecessary alerts [14,127], and as pig health experts, can assist engineers and programmers in deciding on the correct observations, based on biological relevance and validation [128].

### 5.2. Robustness of Sensors

Some are concerned about whether sensors will tolerate on-farm environment assaults such ammonia, dust, moisture, weather conditions or high pressure washing. While off-the-shelf 2D cameras and microphone sensors, used in the security industry, may supply hardware for imagining and sound, the development of newer sensors and their applications will need to be tested within farm conditions. In commercial barns, lighting fluctuations, backgrounds sounds, controlling for pests, or ambient weather conditions might prevent the system from recognizing the subject and accurately determining its features. Open-access development between farmers and developers is a possibility to expose developers to the environment and constraints in which this technology must operate. Practitioners can act as the liaison, allowing the developer to enter the farm, under some agreements, to identify the design constraints that the sensors must satisfy to be robust.

## 6. Limitations and Opportunities

Many of the prior-mentioned applications in research could evolve into commercial products that improve profits for pig producers [15]. Therefore, the adoption of precision livestock farming by farmers will require input from trusted allies to disseminate knowledge and to provide guidance on issues such as business and proprietary relationships with sensor developers, and pertaining to stakeholder traceability.

### 6.1. Data Rights, Transparency and Traceability

Further improvements to animal welfare are needed as consumers demand more transparency of where their meat comes from [129], initiating farm-to-fork concepts and farm assurance programs such as PQA Plus^®^. In addition to this, Blockchain—an incorruptible electronic ledger that can track each transaction of a food item’s journey through the food chain—would increase the transparency of production practices and documentation by farms and data driven management—a pervading issue in (pig) production and among stakeholders [130]. Pharmaceutical companies, regulatory bodies and policy makers will also realize the benefits of improved data collection for global health surveillance [131].

Sensor driven automated data collection, when integrated with suppliers, captures and records multiple attributes for each animal, age, pedigree, growth rates, health, feed conversion rates, meat quality and close-out or kill-out percentage [124]. This data can drive producer profit strategies of shipping or culling animals at optimal time points and minimize the cost and management of therapeutic drug use [59]. An example of an integrated precision livestock farming framework is the IOF2020 [123]. A European Union funded project using sensors is collecting and linking real-time farm data of individual animals/animal groups to data from slaughter plants with the intent to provide farmers with feedback on their management strategies and help to optimize animal well-being and production profits.

Still, continued discussion and policy development to protect data is warranted. While most captured data is not considered sensitive (e.g., a single image of a sow demonstrating nesting behavior), there may be some sensitivity, especially in aggregate. For example, nesting behavior of many sows could be misconstrued as a stress behavior and may initiate privacy concerns and misuse of information outside its context. Adams-Progar et al., [132] provides a summary of security and privacy issues in the chapter “Internet of Cows”.

To enable the benefits of increased transparency and information sharing along the value chain, Shepherd [124] suggests a framework that will create a responsible model to meet the needs of proper data privacy and standardize technology infrastructure to ensure privacy, encryption, security and management. Data rights of farmers used in the development of sensors can be a difficult topic to handle and at minimum should include binding agreements in which farmers and technology developers agree to terms and conditions. Zhou [133] provides a thorough review on risks and solutions to security and privacy pertaining to data. Practitioners can assist their clients and stakeholders to establish industry standards for responsible storage and use of data to avoid infringements of personal data rights. Shepherd provides a thorough discussion of privacy standards in digital agriculture [124].

### 6.2. Stakeholder Advocacy and Collaboration

Working with veterinary, animal science, and producer associations and regulatory agencies, practitioners retain a balanced view of available and emerging resources. Innately there is an obligation of professionals to learn, introduce and/or supervise new technologies [134]. During 2018 annual meetings, both the American Association of Swine Veterinarians (AASV) (aasv.org) and the American Society for Animal Science (ASAS) (asas.org) offered workshops on precision livestock farming.

In spite of an early emphasis of this technology in Europe and while twenty percent of Dutch dairy farmers have sensors for estrus detection, other sensors are not widely adopted [124]. Farmers may be skeptical to implement precision livestock farming for several reasons. Based on experiences from the human wearable sensor market, it is reported that only 5% of that technology is formally scientifically validated [135]. Another reason livestock farmers may be slow to apply precision livestock farming techniques, is because of prior experience with costs associated with purchasing and maintaining new equipment and computer systems, as well as a challenging and time-consuming learning curve for personnel [11]. One more hesitation, and perhaps the largest technical challenge may include lack of broadband internet access in rural areas. Remote capture and transfer of data often requires internet or cellular access. Recently, Microsoft has announced the Airband Initiative to eliminate the US rural broadband gap by 2022 [136]. Nevertheless, it will take time to address connectivity issues.

### 6.3. Emerging Technological Integration

Two areas of emerging technological advances, albeit requiring considerably more developmental research, could be integrated with precision livestock farming devices. The development of 5G communication combined with Massive Multiple Input Multiple Output (Massive MIMO) could assist in wireless sensor networks’ infrastructure (e.g., reducing the number of required wireless network receivers) and making more efficient devices with low power requirements [137]. Another emerging technology is a potential integration with doplar radar technology to provide remote sensing for both respiration and heart rate [138]. These technologies could provide more efficient ways of capturing swine physiology and well-being while reducing network complexity.

### 6.4. Opportunities

Industry opportunities of precision livestock farming include (1) Workforce development: An automated, objective method to monitor and collect data can simplify labor requirements, reduce the inefficiencies of recurring tasks, save time and attract a different genre of stockperson. Precision livestock farming practices may change the way we work, allocating resources of time and energy to the development of predictive treatment regimes and protocols, (2) Animal welfare: a non-invasive, automated system will improve pig welfare and attention via “individual animal approach”. These techniques can convert farms to research facilities that provide real-time outcomes. An example includes measuring pig aggression [139,140] in the presence of environmental enrichment, (3) Consumer acceptance and enhancement of consumer image of the swine industry: The public expects producers to provide individual attention with good human-animal interaction. While we presume that large commercial producers will become early adopters of technology, precision livestock farming can be farm-size neutral. For example, HerdDogg (herddogg.com, Ashland, Oregon) provides yearly remote health and behavior monitoring services for as few as twenty-five pasture-raised animals. Smaller producers often have an opportunity for niche and direct access to consumers, and the story of the individual animal.

Automation of livestock production raises an ethical concern about machine learning replacing human care [141]. Others argue that based on an individual or per animal approach, it will improve connection between producers and pigs [125]. One of the simplest problems, however, is that it is not always clear what data should be collected and why it is relevant. Practitioners collaborating with engineers and technology developers will create products suitable for on-farm use and positive impacts on the welfare of the animals.

## 7. Conclusions

While the world population is expected to reach more than 9 billion in 2050, the World Bank predicts that the increases in demand for meat must be sustained by 90 per cent of existing farmland [142]—intensification is inevitable. By collecting and analyzing vast quantities of data that no person would be able to complete on their own, precision livestock farming can provide producers with information about the welfare of the whole herd as well as individual animals. The continued development of precision livestock farming and the possibilities for interconnection in the pork value chain, ultimately links consumers and farmers. Consumers will make decisions based on farm practices, and farms can make decisions based on consumer practices [124]. The role of the swine practitioner can be somewhere in the connection, helping to navigate precision livestock farming toward prosperity for all—the farmer, the stockperson and the pig.

## Figures and Tables

**Table 1:** A comparison between various sensors and their applications.

Hardware Comparisons			
Sensor Device and Manufacturer examples	Features	Fallbacks	Applications
**Cameras**			
2D (RGB) — Lorax — eYenamic^®^ 3D (RGBD) — Microsoft Kinect^®^ — Intel Realsense^®^ — eYegrow^®^	— Useful for fine positional and color variational data — High precision and lots of data — Remote sensing (non-invasive) — Fast readings (usually 15–60fps) — Individual or group can be analyzed	— Requires filtering to obtain useful information — Performance is dependent on lighting conditions — May require protective covers against environmental elements	— Optical character recognition — Feature extraction — Motion detection — Topology extraction — Animal distribution and activity
**Thermometers**			
Infrared Imaging (IR) — FLIR^®^ — FLUKE^®^ — TESTO 875^®^	— Useful for biological process observation and night vision — High performance in low visibility settings — Remote sensing (non-invasive) — Fast readings (usually 15–60fps)	— Expensive (Mid hundreds to several thousands per unit) — Environmental factors affect readings	— Remote temperature sensing — Low light imaging — Physiological responses (individualized and group)
Thermistors — Integrated in wearable sensors	— Useful for temperature fluctuations — Inexpensive	— Slow to sense changes — Not an off the shelf system	— Contact temperature sensing — Physiological responses
**Microphones**			
— Soundtalks^®^ — PCM Monitor^®^	— Useful for sound/frequency fluctuations —Immediate readings — Inexpensive	— Easily corrupted by noise	— Monitoring periodic physiological process (in pens and/or barns) — Auditory classification
**Accelerometers**			
Exmples of WSN — Remote Insights^®^ — Smartbow^®^	— Useful for motion tracking — Near instantaneous readings — Embedded into wearable sensors used in wireless sensor networks (WSN)	— Requires external processing to obtain displacement and velocity data —Information is relative (not absolute) — Fragile (can break with sow behavior)	— Motion detection/observation (i.e., walking, nesting behavior) —Positional state tracking (i.e., lying, standing)

## Abbreviations

2D	Two Dimensional
3D	Three Dimensional
AASV	American Association of Swine Veterinarians
CSIA	Common Swine Industry Audit
PQA Plus	Pork Quality Assurance Plus
LED	Light Emitting Diode
LF	Low Frequency
Massive MIMO	Massive Multiple Input Multiple Output
PLF	Precision Livestock Farming
QR Code	Quick Response Code
RFID	Radio Frequency Identification
TOF	Time of Flight
UHF	Ultra High Frequency
WSN	Wireless Sensor Network
